# Molecular Mechanisms Responsible for Mesenchymal Stem Cell-Based Treatment of Viral Diseases

**DOI:** 10.3390/pathogens10040409

**Published:** 2021-04-01

**Authors:** Carl Randall Harrell, Biljana Popovska Jovicic, Valentin Djonov, Vladislav Volarevic

**Affiliations:** 1 Regenerative Processing Plant, LLC, 34176 US Highway 19 N Palm Harbor, Palm Harbor, FL 34684, USA; dr.harrell@regenerativeplant.org; 2 Department of Infectious Diseases, Faculty of Medical Sciences, University of Kragujevac, Svetozara Markovica 69, 34000 Kragujevac, Serbia; 3 Institute of Anatomy, University of Bern, 2 Baltzerstrasse, 3012 Bern, Switzerland; valentin.djonov@ana.unibe.ch; 4 Department for Microbiology and Immunology, Center for Molecular Medicine and Stem Cell Research, Faculty of Medical Sciences, University of Kragujevac, 69 Svetozar Markovic Street, 34000 Kragujevac, Serbia

**Keywords:** mesenchymal stem cells, viruses, diseases, therapy, immunomodulation

## Abstract

Mesenchymal stem cells (MSCs) are adult, immunomodulatory stem cells which reside in almost all postnatal tissues. Viral antigens and damage-associated molecular patterns released from injured and infected cells activate MSCs, which elicit strong antiviral immune response. MSC-sourced interferons and inflammatory cytokines modulate the cytotoxicity of NK cells and CTLs, enhance the antigen-presentation properties of DCs and macrophages, regulate cytokine synthesis in CD4+ T helper cells and promote antibody production in B cells. After the elimination of viral pathogens, MSCs produce immunoregulatory cytokines and trophic factors, prevent the over-activation of immune cells and promote tissue repair and regeneration. In this review article, we summarize the current knowledge on the molecular mechanisms that are responsible for the MSC-dependent elimination of virus-infected cells, and we emphasize the therapeutic potential of MSCs and their secretomes in the treatment of viral diseases.

## 1. Introduction

Among the numerous epidemics of infectious diseases that the world is facing, viral infections are undoubtedly the biggest pandemic threat in the recent era [1]. The epidemic outbreaks caused by viruses represents a critical threat to public health, particularly when preventive vaccines and effective antiviral therapies are not available [1,2]. Since December 2019, humankind has been confronted with a new coronavirus disease (COVID-19) caused by severe acute respiratory syndrome coronavirus (SARS-CoV-2), which has infected more than 110 million people worldwide [2,3]. The pandemic of COVID-19 prompted the scientific world to define new approaches for the prevention and treatment of viral diseases, including the development of new vaccines and antiviral drugs [2]. Despite the fact that vaccination is crucially important for the cessation of pandemic outbreaks, the confirmation of safety and efficacy of newly developed vaccines usually need long-term investigation [3]. Accordingly, antiviral drugs are usually used in clinical practice as the first line of defense against life-threatening viral diseases [4]. Antiviral agents are primarily designed to affect one or more phases of the viral life cycle (including viral-host interaction, genome coping and viral maturation) and/or to enhance antiviral immune response by improving the cytotoxicity of natural killer (NK) cells and cytotoxic CD8+ T lymphocytes (CTLs) or by inducing an increased production of antiviral cytokines by dendritic cells (DCs) and CD4+ helper T cells [4]. However, the long-term use of antiviral drugs may provoke the excessive release of inflammatory cytokines from activated immune cells, which could result in the development of life-threatening cytokine storm and massive injury of parenchymal cells [5]. Therefore, new therapeutic agents which may elicit potent antiviral immune response and, at the same time, could prevent the development of systemic and detrimental inflammatory response, could have beneficial effects in the treatment of viral diseases [4].

Mesenchymal stem cells (MSCs) are immunoregulatory stem cells that exist in essentially all adult tissues which orchestrate antiviral immune response [6,7]. Immediately after viral entry, damage-associated molecular patterns (DAMPs) and/or pathogen-associated molecular patterns (PAMPs) induce the generation of pro-inflammatory (MSC1) phenotypes in MSCs [7]. MSC1, through the secretion of inflammatory chemokines, attracts circulating leucocytes into the inflamed tissues and regulate the function of all immune cells that are involved in antiviral immune response (DCs, macrophages, NK cells, B lymphocytes, CD4+ T helper cells and CTLs) [7]. MSC-sourced interferons (IFNs) modulate the cytotoxicity of NK cells and CTLs, enhance the antigen-presentation properties of DCs and macrophages, regulate cytokine synthesis in CD4+ T helper cells and antibody production in B cells, crucially contributing to the efficient removal of virus-infected cells [7,8]. During the remodeling phase of tissue repair, MSCs obtain anti-inflammatory phenotypes and, through the release of immunoregulatory molecules (transforming growth factor-β (TGF-β), indolamine 2,3-dioxygenase (IDO), interleukin (IL)-10, IL-1 receptor antagonist (IL-1Ra), prostaglandin E2 (PGE2)), suppress the excessive activation of immune cells, preventing the generation of cytokine storm and detrimental systemic inflammatory response [8]. Accordingly, due to their potent immunomodulatory characteristics, MSCs are, in large number of experimental studies, explored as potentially new remedy in the treatment of viral diseases [9,10]. In this review article, we summarized current knowledge on the signaling pathways and cellular mechanisms that are involved in the MSC-dependent elimination of virus-infected cells and for MSC-based repair and regeneration of tissues initially injured by viral pathogens. An extensive literature review was carried out in February 2021 across several databases (MEDLINE, EMBASE and Google Scholar), from 1990 to the present. Keywords used in the selection were as follows: “mesenchymal stem cells”, “virus”, “viral infection“, “viral disease”, “immune cells”, “inflammation”, “immunomodulation”, “therapy” and “regeneration”. Experimental studies which emphasized molecular mechanisms responsible for the MSC-dependent modulation of antiviral immune response and clinical trials that provided evidence about efficacy of MSC-based therapy in the treatment of viral diseases were evaluated in this review.

## 2. Molecular Mechanisms Responsible for MSC-Dependent Modulation of Antiviral Immune Response

Cytotoxic NK cells and CD8+CTLs efficiently eliminate infected cells [11]. Infected MSCs express viral antigens on major histocompatibility complex (MHC) class I molecules and directly activate CD8+CTLs [6]. Additionally, upon the activation of Toll-like receptor (TLR)-3, -7 and -9 by viral antigens, MSCs obtain pro-inflammatory (MSC1) phenotypes and produce antiviral cytokines interferon (IFN)-α and IFN-β that enhance the cytotoxicity of CTLs and NK cells [6,9,10]. MSC1-primed CTLs and NK cells produce perforins and granzymes which induce the apoptosis of virus-infected cells by activating BH3-interacting domain death agonist (Bid), pro-apoptotic Bax and/or Bak proteins and caspase-9 and caspase-3 [6,9,10]. Upon activation by MSC1, CTLs and NK cells secrete a large amount of IFN-γ, which enhances the phagocytic properties of tissue-resident macrophages, enabling the efficient removal of apoptotic cells [6]. Additionally, virus-infected MSCs are able to induce the activation of NK cells in a contact-dependent manner, as well [9,10]. An increased expression of UL16-binding protein (ULBP), CD155 and CD112, which are ligands for activating receptors of NK cells (NKp30, NKG2D and CD226, respectively), was observed in infected MSCs, indicating that MSCs may enhance the cytotoxicity and antiviral properties of NK cells in a juxtacrine manner [9,10].

**Figure 1:**
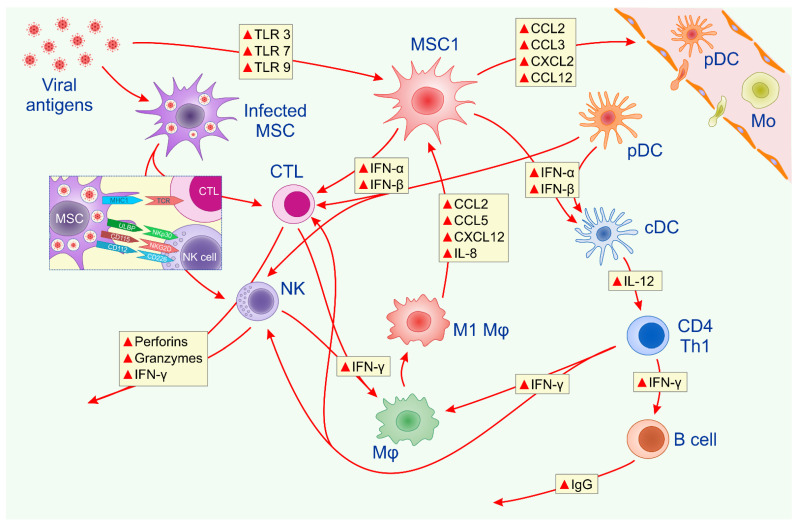
Molecular mechanisms responsible for MSC-based modulation of antiviral immune response. Virus-infected MSCs present viral antigens to CTLs in an MHC class I-dependent manner and express ULBP, CD155 and CD112, which serve as ligands for activation receptors of NK cells. MSC1 secrete antiviral IFN-α and IFN-β that enhance production of perforins, granzymes and IFN-γ in CTLs and NK cells. After sensing viral pathogens in inflamed tissues, MSC1 produces monocyte-attracting chemokines which promote the recruitment of DCs and circulating monocytes into the site of inflammation. MSC1-recruited DCs produce IL-12, IFN-α and IFN-β which induce generation of IFN-γ-producing effector CD4+Th1 cells. CD4+Th1 cells in an IFN-γ-dependent manner promote class-switching in B cells, enabling synthesis and secretion of virus-specific IgG antibodies. CTLs, CD4+Th1 lymphocytes and NK cells produce IFN-γ which induces generation of inflammatory (M1) phenotypes in macrophages, enhancing their antimicrobial properties. M1 macrophages produce MSC-attracting chemokines and cytokines that attract MSC1 from stem cell niches in inflamed tissue, creating a “positive inflammatory loop” which enables efficient elimination of viral pathogens. Abbreviations: mesenchymal stem cells (MSCs); cytotoxic T lymphocytes (CTLs); UL16-binding protein (ULBP); natural killer (NK) cells; interferon (IFN), interleukin 12 (IL-12), dendritic cells (DCs).

After sensing viral pathogens, tissue-resident MSC1 releases monocyte-attracting chemokines which enable the recruitment of circulating monocytes and DCs into the site of inflammation (Figure 1) [6,9]. Plasmacytoid DC (pDC) is characterized by the high constitutive expression of interferon regulatory factor 7 (IRF-7), which plays a crucially important role in the transcriptional activation of virus-inducible cellular genes, including type I interferon genes [12]. MSC-recruited pDCs respond to viruses with a rapid and robust production of IFN-α and IFN-β, which enhances the antiviral properties of CTLs and NK cells [6,9,10]. Following IFN production, pDCs mature into antigen presenting cells that help to shape the adaptive immune response by increasing the expression of MHC class I and II molecules, enabling the activation of virus-specific naïve CD8+ and CD4+ T lymphocytes [12]. IFN-α and IFN-β, derived from MSC1 and pDCs, activate myeloid DCs (mDC) and enhance their antigen-presenting properties, as well [9]. The cross-talk between activated cDC, CTLs and CD4+ T helper lymphocytes is crucially important for an efficient antiviral immune response [11]. IL-12, derived from activated cDCs, induces generation of IFN-γ-producing effector CD4+Th1 cells, which, in an IFN-γ-dependent manner promote, class-switching in B cells, enabling the synthesis and secretion of virus-specific IgG antibodies [11]. Additionally, by producing IFN-γ, CD4+Th1 cells enhance the cytotoxicity of CTLs and NK cells, resulting in the apoptotic cell death of infected cells [11].

CD4+Th1 cell-sourced IFN-γ induces the generation of pro-inflammatory (M1) phenotypes in tissue-resident macrophages, enhancing their phagocytic properties [11]. Inflammatory M1 macrophages efficiently remove apoptotic cells and produce MSC-attracting chemokines that attract MSC1 from stem cell niches in inflamed tissue [13]. Importantly, these M1 macrophage-sourced inflammatory factors enhance the production of antiviral cytokines, IFN-α and IFN-β in recruited MSC1 [14]. Accordingly, an interplay between M1 macrophages and MSC1 creates a “positive inflammatory loop” in the inflamed tissue, which enables the efficient elimination of viral pathogens, apoptotic cells and cellular debris [14].

Upon the removal of virus-infected cells, MSCs attenuate on-going inflammation, prevent the over-activation of immune cells and restore tissue homeostasis [15]. During the resolution phase of tissue repair, under the influence of inflammatory cytokines (tumor necrosis factor alpha (TNF-α) and IFN-γ), MSCs obtain anti-inflammatory (MSC2) phenotypes [6]. MSC2 produces a large amount of anti-inflammatory and pro-angiogenic factors that enhance tissue regeneration after the elimination of virus-infected cells [9,10,15]. MSC2, through the activation of aryl hydrocarbon receptor (AhR), induces the expansion of innate lymphoid cells (ILCs) [16]. Amphiregulin, released from MSC-activated ILCs, promotes the synthesis of proteins that regulate the proliferation of epithelial cells (c-Myc, cyclin D1 and CDK4), importantly contributing to the regeneration of injured epithelial cells [16]. MSC2-derived immunomodulatory molecules (IL-10, PGE2 and galectin-3) induce the generation of tolerogenic phenotypes in DCs [8]. MSC2 and tolerogenic DCs, in an IDO-dependent manner, induce the expansion of immunosuppressive Tregs, enabling the creation of an immunosuppressive microenvironment [8]. Additionally, MSC2-sourced PGE2 and IDO induce the generation of immunosuppressive (M2) macrophages which, in a TGF-β and IL-10-dependent manner, induce tissue repair [17]. Additionally, by producing pro-angiogenic and trophic factors, MSC2 induces neo-vascularization and promotes the proliferation and differentiation of tissue-specific progenitor and stem cells, enabling the enhanced repair and regeneration of tissues, initially injured by viral pathogens [18].

## 3. MSC-Based Therapy of Viral Hepatitis

The beneficial effects of MSCs in the attenuation of acute hepatitis and liver failure have been documented in experimental studies [19,20,21,22]. MSCs, in an NO and IDO-dependent manner, suppress the activation of hepatotoxic, IFN-γ and IL-17-producing CTLs; CD4+Th1 and Th17 lymphocytes and natural killer T (NKT) cells; inflammatory IL-12, IL-1β, IL-6 and IL-23-producing DCs; and TNF-α and IL-1β-producing macrophages in the liver and induce the proliferation of regulatory FoxP3-expressing T and NKT cells [20,21]. Additionally, MSC-sourced HGF promotes hepatocyte proliferation and liver regeneration [8,19].

Similar to these results are findings obtained in recently conducted clinical trials [23,24,25]. As evidenced by Lin and colleagues [23], allogeneic bone marrow-derived MSCs (BM-MSCs) significantly increased the 24-week survival rate of 56 patients suffering from hepatitis B virus (HBV)-related acute on chronic liver failure (ACLF). BM-MSCs (1–10 × 10^5^ cells/kg) were intravenously (i.v.) injected (once per week for 4 weeks). There were no infusion-related side effects, indicating that the i.v. administration of MSCs is a safe approach for the treatment of patients with life-threatening ACLF [23]. Importantly, markedly improved clinical laboratory measurements, including serum total bilirubin and the Model for End-Stage Liver Disease (MELD) score, were observed in MSC-treated patients with HBV-ACLF compared with the patients from the control group who received standard therapy (entecavir combined with nutritional supplementation, human serum albumin and frozen plasma) [23]. Additionally, MSC-based therapy significantly reduced mortality and multiple organ failure in patients with HBV-ACLF [23]. The incidence of severe infections was higher in a group of patients who received standard therapy than in the MSC-treated patients, suggesting that MSCs managed to suppress the activation of hepatotoxic immune cells without causing systemic immunosuppressing and secondary immunodeficiency [23].

Single infusion of umbilical cord-derived MSCs (UC-MSCs; 100 × 10^6^ cells) significantly improved the liver function and survival of entecavir-treated patients with HBV-ACLF [24]. UC-MSC-treated patients had a better appetite, alleviated abdominal distension and relieved fatigue compared to UC-MSC-non-treated patients with HBV-ACLF [24]. UC-MSCs significantly enhanced hepatocyte function as indicated by improved liver functional markers, including albumin, alanine aminotransferase, aspartate aminotransferase, total bilirubin, direct bilirubin, prothrombin time (PT) and international normalized ratio (INR) [24]. Additionally, a significantly decreased MELD score was noticed in UC-MSC-treated patients with HBV-ACLF, 4 weeks after UC-MSC infusion [24]. Importantly, two years of follow-up revealed that UC-MSCs significantly increased the survival of entecavir-treated patients with HBV-ACLF without causing severe side effects, suggesting that the infusion of UC-MSCs could be considered as an adjunctive therapy to the standard of care treatment for patients with HBV-ACLF [24].

UC-MSC-sourced exosomes (UC-MSC-Exos) significantly improved the beneficial therapeutic effects of IFN-α or telaprevir, which are usually used as the standard therapy for patients suffering from hepatitis C virus (HCV) infection [25]. MSC-Exos contain all immunosuppressive and angiomodulatory factors as their parental MSCs [26]. As evidenced by Qian et al. [25], UC-MSC-Exos contain several miRNAs (let-7f, miR-145, miR-199a and miR-221) which bind to the HCV RNA and prevent the replication of HCV. Additionally, UC-MSC-Exos showed synergistic effects with IFN-α or telaprevir in the suppression of HCV replication and, therefore, could be considered as potentially new adjuvant therapeutic agents in the treatment of patients with HCV [25].

## 4. MSC-Based Therapy of Difficult-to-Treat Patients with HIV

The progressive loss of CD4+ T cells increases the risk of opportunistic infections in patients with HIV [27]. Highly active antiretroviral therapy (HAART) is very effective in the restoration of CD4+T cells [28]. However, about 20% of HAART-treated patients fail to achieve sufficient reconstitution of CD4+ T lymphocytes and are considered as immune non-responders (INRs) [29]. HIV-infected INRs experience an increased risk of opportunistic infections and shorter life expectancy and, therefore, treatment of these patients is among the most important challenges which needs to be solved [29].

Zhang and colleagues were the first to show that UC-MSC-based therapy may efficiently improve host immune reconstitution in HIV-infected INRs and proposed that the combination of UC-MSCs and HAART could be used as a novel therapeutic approach for INR patients [30]. INR patients with HIV who had been receiving HAART for at least 12 months were randomly assigned to the experimental (n = 7) or control group (n = 6) to intravenously receive HAART and UC-MSCs (0.5 × 10^6^/kg body weight; 1 infusion/month for 3 months) or HAART and saline, respectively [30]. Importantly, UC-MSCs did not provoke life-threatening immunosuppression in HAART-treated INRs but altered the ratio between naïve and effector T helper cells [30]. The total number of naive and central memory CD4+ T lymphocytes was significantly increased in UC-MSC-treated INRs, while effector and effector memory CD4+ T cells were not expanded by UC-MSCs [30]. Importantly, the significantly enhanced production of IL-2, which is crucially responsible for the proliferation of CD4+ T cells, was observed in peripheral blood mononuclear cells of UC-MSC-treated INRs after their in vitro re-activation by HIV antigens [30]. Additionally, the down-regulated expression of PD-1, which is associated with HIV-specific T-cell exhaustion, was observed on the membranes of CD4+ T cells of UC-MSC-treated INRs, indicating that UC-MSCs prevented the PD-1-dependent apoptosis of CD4+ T cells [30]. Importantly, the UC-MSC-induced reconstitution of CD4+ T cells was accompanied by attenuated systemic inflammatory response, as evidenced by significantly reduced levels of D-dimer, CRP, TNF-α, IL-6 and IL-9 in UC-MSC-treated INRs. The mechanisms responsible for the UC-MSC-dependent suppression of systemic inflammation are unclear, but it may be attributed to the immunosuppressive activity of Tregs, since a significantly higher number of Tregs was observed in the peripheral blood of UC-MSC-treated INRs and was not seen in saline-treated controls [30]. Although these findings are promising, it should be noted that all conclusions were made based on the results obtained in only seven HIV-infected INRs [30]. Accordingly, a large-scale randomized study should be realized in the near future to confirm the beneficial effects of UC-MSCs in the therapy of difficult-to-treat patients with HIV.

## 5. MSCs and Their Secretomes as Potentially New Therapeutic Agents in the Treatment of SARS-CoV-2-Induced Lung Inflammation

The SARS-CoV-2-induced infection of pneumocytes and ciliated cells of the airways usually results in alveolar injury and lung inflammation [3]. In the majority of COVID-19 patients, alveolar macrophages, lung-infiltrated DCs and T cells efficiently eliminate the virus and prevent disease progression [3]. However, severe cytokine storm might develop in some patients due to the massive production of inflammatory cytokines and chemokines by SARS-CoV-2-over-activated immune cells [31]. The excessive secretion of these inflammatory mediators results in the development of severe, life-threatening pneumonia, lung edema and acute respiratory distress syndrome (ARDS) [31].

**Figure 2:**
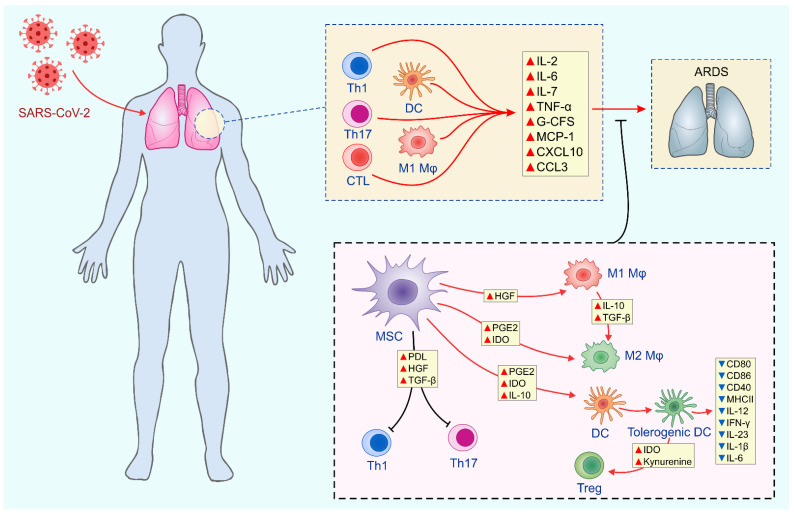
MSC-based treatment of SARS-CoV-2-induced ARDS. Severe cytokine storm might develop in patients with SARS-CoV-2 due to the massive production of inflammatory cytokines and chemokines by SARS-CoV-2-over-activated DCs, M1 macrophages, CTLs and CD4+Th1 and Th17 lymphocytes. MSC-sourced HGF, IL-10 and TGF-β act synergistically to induce generation of alternatively activated, anti-inflammatory (M2) phenotypes in alveolar macrophages. MSC-derived PGE2, IL-10 and IDO generate immunosuppressive phenotypes in lung DCs and induce generation of immunosuppressive Tregs. MSCs induce PDL-dependent apoptosis of inflammatory T cells, reducing their presence in injured lungs. Additionally, MSC-sourced TGF-β and HGF induce the G1 cell cycle arrest and prevent proliferation of activated Th1 and Th17 cells. Abbreviations: mesenchymal stem cells (MSCs); severe acute respiratory syndrome coronavirus (SARS-CoV-2); dendritic cells (DCs); cytotoxic T lymphocytes (CTLs); hepatocyte growth factor (HGF); interleukin 10 (IL-10); transforming growth factor beta (TGF-β); program death ligand (PDL); prostaglandin E2 (PGE2) indolamine 2, 3 dioxygenase (IDO).

MSCs may prevent the development of SARS-CoV-2-induced lung injury and ARDS by inducing the generation of immunosuppressive phenotypes in lung-infiltrated inflammatory cells (Figure 2) [32]. MSC-sourced hepatocyte growth factor (HGF), IL-10 and TGF-β act synergistically to induce the generation of alternatively activated, anti-inflammatory (M2) phenotypes in alveolar macrophages [33]. MSC-derived PGE2, IL-10 and IDO generate tolerogenic phenotypes in lung-infiltrated DCs and induce the generation of immunosuppressive Tregs [8]. MSCs may directly suppress the expansion of inflammatory, IFN-γ-producing Th1 and IL-17-producing Th17 cells in the injured lungs [34]. MSCs, in a program death ligand (PDL)-dependent manner, induce the apoptosis of over-activated T cells, alleviating their detrimental effects on the inflamed lungs [33]. In addition, MSC-sourced TGF-β and HGF cause the G1 cell cycle arrest and inhibit proliferation of activated Th1 and Th17 cells by suppressing the activation of the Jak-Stat signaling pathway [8].

In addition to their immunoregulatory characteristics, the beneficial effects of MSCs in attenuation of SARS-CoV-2-induced lung injury could be attributed to their angio-modulatory properties, as well [32,33]. After engraftment in ischemic tissues, MSCs produce pro-angiogenic factors (vascular endothelial growth factor (VEGF), PDGF, angiopoietin-1 and placental growth factor), which induce the proliferation of endothelial cells, preventing ischemia-induced injury [33].

Since MSCs may efficiently suppress detrimental immune response and are able to provide additional oxygen supply to injured lungs, several clinical trials have investigated the therapeutic potential of MSCs in the treatment of SARS-CoV-2-induced lung inflammation [35,36,37,38].

Leng and colleagues showed that the intravenous infusion of MSCs (1 × 10^6^ cells/kg) attenuated detrimental immune response in the inflamed lungs and improved respiratory function in 10 patients with SARS-CoV-2 [35]. Within 48 to 96 h after MSC infusion, the oxygen saturation significantly increased, and pneumonia-related symptoms (high fever, shortness of breath, and cough) disappeared in all of MSC-treated patients [35]. A computed tomography (CT) confirmed the beneficial effects of MSCs [35]. SARS-CoV-2-related ground-glass opacity disappeared after MSC infusion [35]. Importantly, MSCs prevented the influx of inflammatory immune cells in the lungs of patients with COVID-19 and favored the expansion of anti-inflammatory and regulatory cells, attenuating on-going inflammation [33,35]. MSCs prevented the development of detrimental systemic inflammatory response, as evidenced by a significant decrease in the plasma levels of C-reactive protein (CRP) and TNF-α upon MSC injection [35]. Additionally, MSCs completely restored liver and kidney function and prevented the development of SARS-CoV-2-induced multiple organ dysfunction [35].

Hashemian and colleagues conducted a phase 1 clinical trial to evaluate the safety, tolerability and efficacy of the multiple infusions of placenta-derived MSCs (PL-MSCs) and UC-MSCs in the treatment of critically ill patients with COVID-19 [36]. A total of 11 patients with SARS-CoV-2 with life-threatening hypoxemia and ARDS who required artificial respiratory support intravenously received PL-MSCs or UC-MSCs (200 × 10^6^ cells/infusion; total of three infusions) [36]. Significantly reduced dyspnea, improved oxygenation, down-regulated serum levels of inflammatory cytokines (TNF-α, IL-8 and IL-6) and CRP were observed in six MSC-treated patients [36]. Five MSC-treated patients were discharged from the intensive care unit (ICU) 2–7 days after MSCs infusion, while one patient was discharged from the ICU on day 18. Four MSC-treated patients who had signs of multiorgan failure died within 5–19 days after the first MSCs injection, and one patient developed cardiac arrest on day 7 of the MSC infusion [36]. Although results obtained in this study indicated that multiple infusions of MSCs may rapidly improve respiratory function and reduce systemic inflammation in patients with COVID-19, the fact that beneficial effects were not observed in 45% (5/11) of MSC-treated patients suggested the need of a new, larger clinical trial, which should delineate the efficacy of MSC-based therapy in patients suffering from severe COVID-19-related lung injury [36].

Accordingly, Shi and coworkers performed a phase 2 clinical trial to assess the efficacy of UC-MSCs in the treatment of severe SARS-CoV-2-induced ARDS [37]. Patients with COVID-19 were randomly assigned to the experimental (n = 65) and control groups (n = 35) to receive either UC-MSCs (4 × 10^7^ cells per infusion) or placebo on days 0, 3 and 6 [37]. UC-MSCs reduced lung lesion volume, increased the resolution of lung solid component lesions and significantly improved the respiratory function of patients with COVID-19 [37]. These beneficial effects were not noticed in placebo-treated patients [37]. The 6-min walk test (6-MWT), performed on the 28th day after the onset of treatment, showed an increased distance in patients treated with UC-MSCs, indicating the UC-MSC-based restoration of lung function [37]. These promising results indicate that the use of UC-MSC could be considered as an adjunctive therapy to the standard of care treatment for patients with COVID-19 [37]. However, it should be noted that a phase 3 clinical trial is still required to further evaluate UC-MSC-based effects on mortality and long-term pulmonary disability.

Sengupta and colleagues demonstrated the efficacy of MSC-Exos in the therapy of SARS-CoV-2-induced moderate-to-severe ARDS [38]. The infusion of BM-MSC-Exos resulted in an improvement of respiratory function and restoration of peripheral blood leucocytes in 83% of patients with SARS-CoV-2 [38]. However, despite these promising results, it should be noted that life-threatening lung inflammation and ARDS were observed in 12.5% (3/24) of MSC-Exo-treated patients with SARS-CoV-2, indicating that the beneficial effects of MSC-Exos must be confirmed in larger clinical trials.

## 6. Safety Issues Related to the Clinical Use of MSCs in the Treatment of Viral Diseases

MSCs may be easily isolated from almost all adult tissues [6]. MSCs do not alter their phenotype and function after long-term passaging; can be expanded in vitro; and, in an appropriate number, can be transplanted in patients that need MSC-based therapy [6,7,8]. Although the transplantation of MSCs has been used as a new therapeutic approach for the treatment of inflammatory disorders [39], the fact that MSCs could be targeted by viruses [9,10] raises serious safety concerns for their use in clinical settings.

MSCs become functionally defective following HIV and herpes virus infection [40,41]. HIV infection suppressed the proliferation and increases the production of pro-inflammatory cytokines (TNF-*α*, IL-1β and IL-6) in MSCs [40], while cytomegalovirus (CMV) infection down-regulates IDO production in MSCs, attenuating their immunomodulatory properties [41].

HIV and herpes viruses could incorporate their genetic information into the DNA of MSCs, and, therefore, MSCs could serve as reservoirs for these pathogens [42,43,44]. HIV infection impairs proliferation and attenuates the immunosuppressive properties of MSCs [42]. Rollin and colleagues showed that MSCs could transmit parvovirus B19 to hematopoietic cells [43], while Soland and co-workers reported that 7 of 19 “healthy” CMV-seropositive donors of BM-MSCs harbored low copy numbers of CMV DNA [44].

In contrast to HIV and herpes viruses, it seems that SARS-CoV-2 does not infect MSCs [33]. RNA-sequencing analysis showed that MSCs did not express angiotensin-converting enzyme 2 (ACE2) and transmembrane protease serine 2 (TMPRSS2) receptors, which are crucially important for the interaction of SARS-CoV-2 and target cells [35]. Accordingly, most probably, MSCs are not permissive for SARS-CoV-2 and could not serve as reservoirs for this virus [33].

There are contradictory findings regarding the susceptibility of MSCs to HBV infection [45,46,47,48]. As demonstrated by Ma and colleagues [45], BM-MSCs obtained from healthy donors fully supported HBV replication and secretion, making them a potential reservoir of HBV. These findings were supported by Rong and co-workers, who demonstrated that HBV antigens and HBV DNA were detected in MSCs and that intravenously injected HBV-exposed MSCs were able to harbor and transport HBV to the injured tissues [46]. In contrast to these data are results obtained by Xie et al. [47] and Wang et al. [48], who isolated MSCs from the BM and AT of patients with HBV and found that both BM-MSCs and AT-MSCs were resistant to HBV infection. However, it should be noted that MSCs from chronic patients with HBV were not able to optimally proliferate [49], suggesting that the therapeutic potential of autologous MSCs in the treatment of patients with HBV should be carefully explored in future randomized clinical trials.

Due to the reduced expression of MHC I and II molecules, MSCs have been considered as “hypoimmunogenic” cells which could be used in allogeneic transplantation [6]. Nevertheless, the injection of allogeneic MSCs could activate adaptive immunity in MHC-mismatched recipients, which resulted in the severe aggravation of autoimmune and chronic inflammatory diseases [50]. Elevated serum levels of Th1 and Th17 cell-sourced inflammatory cytokines (IFN-γ, IL-17 and IL-22), an increased number of peripheral blood CD4+ T helper and CD8+CTLs and the aggravation of inflammatory diseases were observed in MSC-treated patients that received allogeneic MSCs [50], raising safety concerns related to the use of allogeneic MSCs in clinical settings.

## 7. Conclusions and Future Perspectives

MSCs orchestrate antiviral immune response crucially contributing to the elimination of infected cells. [9,10] During the early phase of viral infection, MSCs, activated by viral antigens, elicit strong immune response by producing pro-inflammatory cytokines which enhance the antiviral properties of immune cells. After the elimination of viral pathogens, MSCs produce immunoregulatory cytokines and trophic factors that support the repair and regeneration of injured tissues [9,10].

Despite these promising findings, it should be noted that MSCs express receptors which are used by HIV, HBV and herpes viruses for their interaction with target cells [42,43,44]. Therefore, MSCs are permissive for these viruses and could transmit them in allogeneic recipients [42,43,44]. Accordingly, prior to transplantation, donor MSCs must be screened for the presence of HIV, HBV and herpes viruses in order to prevent the incidence of viral-associated diseases and to assure the safety of MSC-based therapy.

Since the vast majority of MSC-based therapeutic effects in the treatment of viral diseases are attributed to the activity of MSC-derived factors [9,10], MSC-sourced secretomes, particularly MSC-Exos, are considered as potentially new adjuvant therapeutic agents in the treatment of viral diseases [25]. However, several challenges need to be addressed before the clinical use of MSCs-Exos. Bearing in mind that MSC-Exos are highly heterogeneous depending on the tissue origin of the MSCs from which they were derived, the pre-selection of the most effective tissue source of MSCs-Exos could be of crucial importance for their efficacy [17]. Additionally, the injection frequency and the exact therapeutic dose which maintain the long-lasting effects of MSC-Exos should be defined for each MSC-Exo-treated viral disease [26]. Finally, the safety issues related to the MSC-Exo-based transmission of viral DNA must be carefully evaluated in future studies before MSC-Exos can be widely offered as new antiviral therapeutic agents.

## Data Availability

Not applicable.
